# How do night-time awakenings, assistive technologies, and help-seeking behaviors impact the sleep of Australian carers? A cross-sectional study

**DOI:** 10.1186/s12877-026-07024-6

**Published:** 2026-02-09

**Authors:** Hannah Commins, Grace E. Vincent, Madeline Sprajcer, Rosemary Gibson, Kate Vincent, Spencer S. H. Roberts, Alexandra E. Shriane, Patrick J. Owen

**Affiliations:** 1 Appleton Institute and School of Health, Medical and Applied Sciences, Central Queensland University, 44 Greenhill Road, Wayville, Adelaide, South Australia 5034 Australia; 2 Institute for Physical Activity and Nutrition, Deakin University, Geelong, Victoria Australia; 3 School of Psychology, Massey University, Palmerston North, New Zealand; 4 Flinders Medical Centre, Flinders University, Adelaide, South Australia Australia; 5 Eastern Health Emergency Medicine Program, Melbourne, Victoria Australia; 6 Eastern Health Clinical School, Monash University, Melbourne, Victoria Australia

**Keywords:** Caregiving, Support devices, Assistance, Technology, Help

## Abstract

**Background:**

Informal carers may experience poor sleep outcomes due to the demands of their role. This study explored the association between the number of night-time awakenings, assistive technology use, and help-seeking behaviors on the sleep quality of adult carers.

**Methods:**

An online quantitative, cross-sectional survey design was utilised. Participants (*n* = 152) included Australian carers aged 18 to 64 years who obtained inadequate sleep hours (< 7 h per night).

**Results:**

Carers who reported more than two awakenings per night (48.5%) had poorer sleep quality, as measured by the Pittsburgh Sleep Quality Index, when compared to carers who reported no awakenings (η² = 0.06). The use of assistive technology was not associated with improved sleep quality (Cohen’s d = − 0.04). Carers who reported help-seeking for sleep problems also reported poorer sleep quality compared to carers who had not sought help (Cohen’s d = 0.67).

**Conclusion:**

These findings indicate that despite the availability of both assistive technologies and various help-seeking sources (e.g., general practitioners, psychologists), carers still experience poor sleep. With an aim to reduce carer burden, future research should investigate ways to decrease night-time awakenings for carers, examine the effect of care-recipient condition on the relationship between assistive technology use and carer sleep quality, and employ a longitudinal study design to evaluate the efficacy of help-seeking strategies for carer sleep.

**Supplementary Information:**

The online version contains supplementary material available at 10.1186/s12877-026-07024-6.

## Introduction

As healthcare systems worldwide face considerable strain to provide support to those in need of daily assistance, informal carers provide necessary yet often undervalued relief [1]. Informal care refers to the provision of unpaid care to someone, often a family member or friend, who requires marked assistance due to disability, medical or mental health issues, or age-related health complications [2, 3]. In Australia, it would cost almost $80 billion per year to replace all informal care with paid workers [4]. The true weight of the burden placed on carers is frequently overlooked, with carers often being required to be on-call to meet caregiving needs at any time of the day or night [5]. As a result, informal care has widely been associated with negative sleep outcomes for carers [5–7]. This issue is especially important in the context of population ageing, with a substantial proportion of informal carers supporting older adults experiencing age-related decline or chronic health conditions. Therefore, understanding sleep quality and related help-seeking behaviours among carers has direct relevance for improving health outcomes in ageing populations.

Obtaining adequate sleep is a fundamental determinant of health [8], with adequate sleep for adults defined as obtaining between 7 and 9 h per night [9]. Inadequate sleep is associated with marked short- and long-term health complications, including decreased quality of life, emotional distress, and increased risk of developing chronic illnesses [8, 10]. A meta-analysis of 35 studies (3268 participants) showed that carers obtained 2.4 to 3.5 h less sleep per week when compared to age-matched non-carers (Hedges’ g = -0.29; 95% CI, -0.48 to -0.09) [11]. Multiple factors create an especially challenging set of circumstances that impact the sleep of carers, including immense physical, psychological, and financial burdens [2, 12]. In addition to inadequate sleep duration, recent research has identified that carers are at risk of negative health outcomes such as fatigue, increased stress, and poor mental health due to poor sleep quality [13]. Sleep quality has been characterised by using related, yet distinct, measures, including: sleep efficiency, sleep latency, sleep duration, and wake after sleep onset [14]. Poor sleep quality has widely been recognised as a contributing factor to negative health outcomes and disease such as diabetes, cardiovascular disease, obesity, depression, and anxiety [14]. Overnight awakenings to provide care (e.g., assisting with toileting, administering medication) may greatly impact the sleep quality of carers [5, 8], as night time arousal correlates with poorer subjective sleep quality in the general population [5, 8, 15, 16]. For example, among young carers aged 15–24 years, self-reported sleep quality was poorer for carers who reported two or more night-time awakenings than for those who experienced less than two awakenings [5]. However, the relationship between night-time awakenings and sleep quality is yet to be explored within a sample of adult carers.

In an effort to reduce the burden of caregiving demands overnight, carers may choose to use assistive technologies (i.e., devices aiming to improve the independence, safety, or overall wellbeing of a care-recipient) [17, 18]. For example, the use of continuous glucose monitors may reduce the need for manual overnight activities (i.e., waking to assess blood glucose levels). Some carers, however, report an increased number of awakenings due to device alerts [19]. Due to conflicting findings regarding the effect of assistive technology on carer sleep, this is an area which demands further investigation.

Many carers may require help to improve their sleep [5, 11]. It has been demonstrated, however, that carers often do not seek help until they have reached the point of ‘exhaustion’ [20]. Help-seeking refers to the intentional pursuit of assistance, whether this be via formal or informal systems [21]. Despite the availability of many evidence-based treatment options for improving sleep (e.g., cognitive behavioral therapy, relaxation therapy, sleeping medications), carers’ sleep problems are often undertreated or disregarded [22–24]. Research has reported that carers may be reluctant to seek help as they believe the focus of formal support should be on the care-recipient and that help-seeking for themselves would deter from this goal [25].

This study aims to address these identified gaps in the literature by testing the following hypotheses:

H1 (night-time awakenings and sleep quality):

- H1.1: a higher number of night-time awakenings (i.e., providing overnight care) is associated with poorer sleep quality in adult carers.
- H1.2: a higher number of night-time awakenings (i.e., providing overnight care) is associated with poorer sleep quality in adult carers even after adjusting for age, gender, and the number of care-recipients.
- H1.3: a higher number of night-time awakenings (i.e., providing overnight care) is associated with poorer sleep quality in adult carers even after adjusting for assistive technology use and help-seeking behaviors.

H2 (assistive technology use and sleep quality):

- H2.1: greater use of assistive technology is associated with better sleep quality in adult carers.
- H2.2: greater use of assistive technology is associated with better sleep quality in adult carers after adjusting for age and gender.
- H2.3: greater use of assistive technology is associated with better sleep quality in adult carers after adjusting for night-time awakenings and help-seeking behaviors.

H3 (help-seeking behaviors and sleep quality):

- H3.1: more frequent help-seeking behaviors are associated with better sleep quality in adult carers.
- H3.2: more frequent help-seeking behaviors are associated with better sleep quality in adult carers after adjusting for age and gender.
- H3.3: more frequent help-seeking behaviors are associated with better sleep quality in adult carers after adjusting for night-time awakenings and assistive technology use.

## Methods

### Study design

The current study used a quantitative, cross-sectional methodology, with methods informed by STROBE guidelines (see Supplementary Material A for the STROBE checklist) [26], involving a pre-planned secondary analyses of a larger dataset [27]. Ethics approval for the present study was obtained from Central Queensland University Human Research Ethics Committee (approval number: 22134).

### Setting

A convenience sampling method was utilised; recruitment of participants was facilitated by Carers Australia who distributed the online survey via their own mailing lists. The anonymous survey was hosted on the Qualtrics platform (Provo, UT, USA) and responses were recorded between January and March 2020. Participation in the online survey took approximately 20 min.

### Participants

Inclusion criteria stipulated that participants must: currently provide assistance to someone with a long-term health condition, disability, or age-related impairments, experience inadequate sleep (defined as regularly obtaining less than 7 h of sleep [9], dissatisfaction with their sleep, or use of sleep management strategies), be an Australian resident, and be aged between 15 and 64 years old. If participants did not meet inclusion criteria, they were redirected to the end of the survey and their response was not recorded. Data from participants aged < 18 years is not presented in this study and is presented in an earlier report [5]. See Supplementary Material B for the complete survey content.

### Procedure

Study information and a link to the survey was sent to potential participants, by email, by Carers Australia. Participants were presented with an information sheet which outlined that the survey content would cover their caring duties and sleep experiences and outlined the voluntary and anonymous nature of the survey. No incentives were offered for participation. Informed consent was confirmed via a ‘next’ button which indicated that the participant had understood the information and wished to participate.

### Measures

The survey content was separated into 11 sections with the following seven sections being relevant to the present study: demographics, carer screening, sleep screening, impact of caring on sleep, sleep behavior, sleep quality, and help-seeking. Demographic information included gender, age, number of care recipients, geographical area (major city, inner regional, outer regional, remote, or very remote), regular sleep duration (hours), and satisfaction with current sleep pattern measured using a 5-point Likert scale (very satisfied, satisfied, moderately satisfied, dissatisfied, or very dissatisfied).

### Overnight awakenings

To determine the number of awakenings experienced, participants were asked to indicate how many times, on a typical night, they would be required to wake to provide care.

### Assistive technology use

To gather information on assistive technology use, a broad definition was applied, with participants asked whether they use any assistive technologies or devices, selecting all that applied: bell, care dog, insulin pump, continuous glucose monitor, blood glucose meter, location/movement monitoring system, enuresis monitor, lock/s, temperature controls, fall detector, epileptic seizure alarm, video display/hands free phone, pressure mattress/bedding, personal alarm call system, and other (free-text specify).

### Help-seeking behaviors

Participants were asked to indicate whether they had sought help for their sleep problems within the last 12 months. If the participant had sought help, they were asked to identify the type of help they had sought, selecting all that applied: intimate partner, friend, parent, other relative/family member, mental health professional, general practitioner, allied health professional, psychiatrist, medical specialist, pharmacist, naturopath/homeopath, web/internet browsing, E-book(s), book(s), pamphlets or leaflets, and other (free-text specify). Participants were asked to rate the efficacy of each help-seeking behavior they had sought on a 0-100 scale, where 0 indicated not at all effective and 100 indicated extremely effective.

### Sleep quality

Participants completed the 19-item Pittsburgh Sleep Quality Index (PSQI), which assesses seven subscales: self-reported sleep quality, sleep duration, sleep latency, sleep disturbances, habitual sleep efficiency, use of sleep medication, and daytime dysfunction over the past month [15]. Each subscale is scored using a 4-point Likert scale (0–3 points) to produce a global PSQI score between 0 and 21, with a higher score indicating poorer subjective sleep quality [5, 15]. A global PSQI score < 5 is indicative of ‘good’ sleep quality, while scores of ≥ 5 are indicative of ‘poor’ sleep quality [15]. The PSQI has acceptable test-retest reliability, good criterion and convergent validity, and a high degree of internal consistency (Cronbach’s α = 0.83) [15, 28].

### Data handling

In accordance with the inclusion criteria for the present study, all responses from participants < 18 and > 64 years were removed from the dataset. Older adult responses were removed in order to keep the survey sample limited to adult carers. Due to limited participant numbers, five age groups were formed (18–24 years, 25–34 years, 35–44 years, 45–54 years, and 55–64 years).

For the purposes of analyses, each of the independent variables were binned to create categorical data: the average number of awakenings experienced per night was grouped into three categories: “none”, “1–2”, or “>2”; assistive technology use was grouped into two categories: “no assistive technology use” or “assistive technology use”; and help-seeking behaviors were grouped into two categories: “no help-seeking” or “help-seeking”.

Average number of awakenings were binned into three groups in accordance with prior carer research by Paterson et al. [5], who investigated night-time awakenings in a sample of young carers. A global PSQI score was calculated for each participant who completed the full PSQI; this score was used as a continuous dependent variable.

### Statistical analyses

All analyses were performed using IBM SPSS Statistics for Windows (28, IBM Corp, Armonk, United States of America). Associations between independent variables (overnight awakenings, assistive technology use, help-seeking behaviors) and sleep quality were examined via independent t-test (two groups) or analysis of variance (ANOVA) with Tukey post-hoc adjustment (three or more groups). Estimated marginal means are reported. Sensitivity analyses were performed via analysis of covariance with Tukey post-hoc adjustment controlled for age, gender, and number of care-recipients [29], as well as other independent variables. An α of 0.05 was adopted for all analyses.

## Results

### Participants

A total of 262 eligible carers commenced the survey, with 152 participants completing the full PSQI. Participants tended to be female, aged 45–54 years, living in major cities, and providing care for one person. A summary of demographic characteristics is shown in Table 1.

**Table 1:** Summary of demographic characteristics of Australian adult carers

Participant characteristic	Total (*n* = 262)	
	*n*	%
What is your gender?		
Male	27	10.3
Female	233	88.9
Other	2	0.8
What is your age? (years)		
18–24 years	43	16.4
25–34 years	18	6.9
35–44 years	62	23.7
45–54 years	87	33.2
55–64 years	52	19.8
What best describes the area you live in?		
Major city	111	42.4
Inner regional	87	33.2
Outer regional	52	19.9
Remote	9	3.4
Very remote	3	1.1
How many people do you provide assistance for?		
1	190	72.5
2	54	20.6
3	9	3.4
4	4	1.6
> 4	5	1.9

Note: *n* = number of participants, % = percentage of participants

A summary of participant sleep characteristics is provided in Table 2. Few participants (*n* = 32, 12.6%) regularly obtained 7–9 h of sleep per night, with the majority (86.6%) reporting obtaining less than 7 h of sleep on average. Most participants reported being either dissatisfied or very dissatisfied with their current sleep (76.3%, *n* = 200), with most also reporting regularly experiencing at least one awakening per night to provide care (88.5%, *n* = 232). Carers overwhelmingly reported poor subjective sleep quality, with 151 (99.3%) scoring a global PSQI score ≥ 5, indicating poor sleep quality (*M* = 12.08, *SD* = 3.77) [15].

**Table 2:** Sleep characteristics of Australian adult carers

Sleep characteristic	Total(n=262)	
	n	%
Sleep quality (PSQI score)^a^		
Good sleep (<5)	1	0.7
Poor sleep (≥5)	151	99.3
How many times do you typically need to wake to provide care?		
No overnight awakenings	30	11.5
1-2 overnight awakenings	105	40
>2 overnight awakenings	127	48.5
How many hours of sleep do you regularly get each night?		
≤ 4h	32	12.2
4 to <5h	54	20.6
5 to <6h	70	26.7
6 to <7h	71	27.1
7 to <8h	20	7.6
8 to <9h	12	4.6
≥ 9h	3	1.2
How satisfied/dissatisfied are you with your sleep pattern?		
Very satisfied	1	0.4
Satisfied	4	1.5
Moderately satisfied	57	21.8
Dissatisfied	109	41.6
Very Dissatisfied	91	34.7

Note: n Number of participants, % Percentage of participants, *PSQI* Pittsburgh Sleep Quality Index

^a^A subset of participants (n=156, 59.5%) completed all PSQI subscales to allow for a global score to be calculated

### H1 (night-time awakenings and sleep quality)

**Fig. 1:**
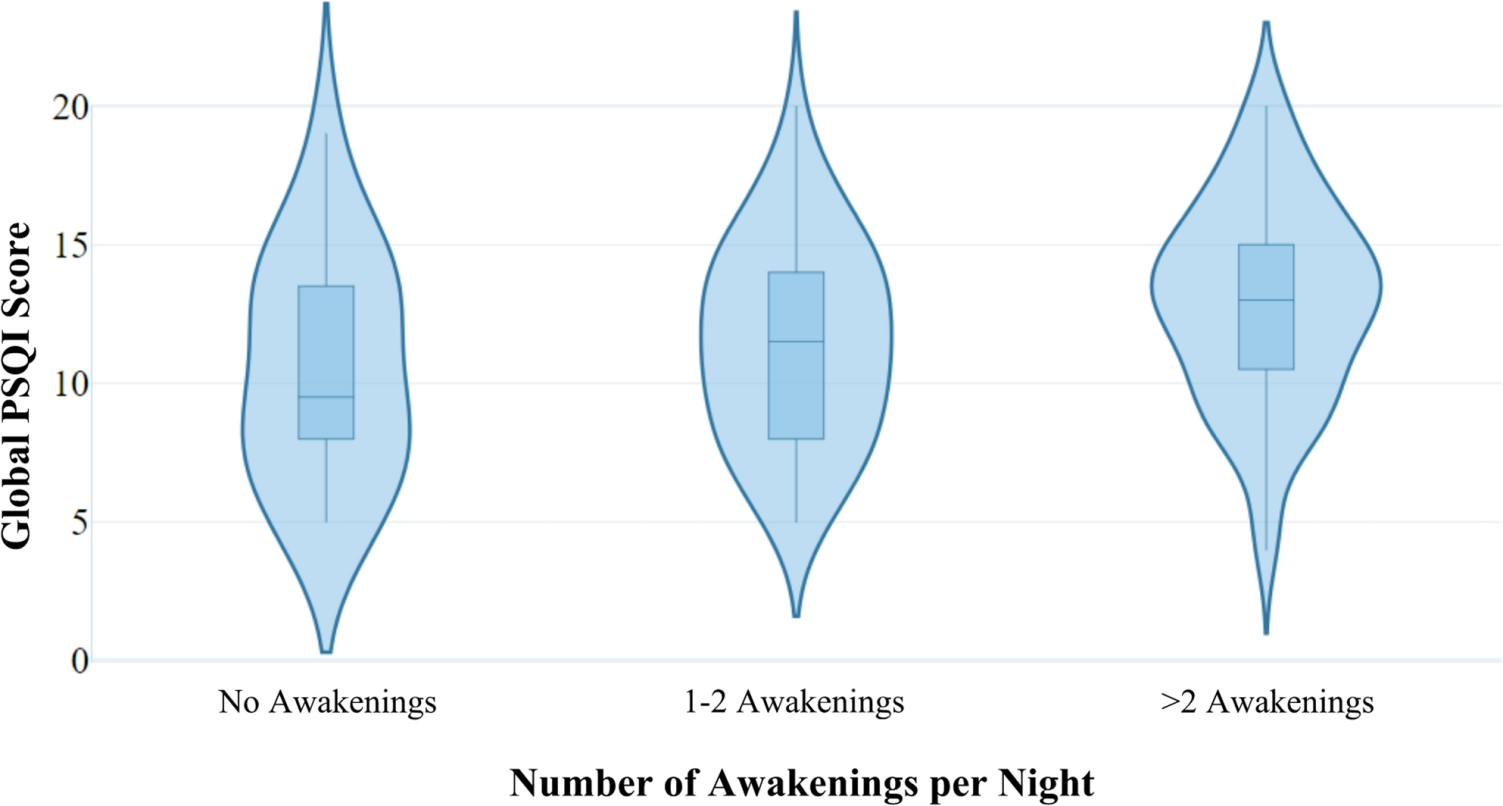
Adult carer global PSQI score by number of awakenings per night. NB: Violin plots showing the global PSQI score of carers across the number of self-reported awakenings. The width of each violin plot represents the approximate frequency of data located in each area. The boxplot within each violin plot shows how data is distributed. The horizontal line within each box plot represents the median value. The box represents the 25th to 75th percentiles and the vertical lines represent the 5th and 95th percentiles

This section examines whether the number of night-time awakenings to provide care is associated with carers’ sleep quality, and whether this relationship persists after adjusting for demographic and care-related factors. There was an observed difference in average PSQI score as a function of the mean number of awakenings to provide care each night, as per Fig. 1. Carers who experienced no awakenings scored the lowest, on average, on the PSQI (*M* = 10.5, *SD* = 4.0), while those who reported > 2 awakenings had the highest PSQI scores on average (*M* = 12.9, *SD* = 3.6).

Difference in PSQI scores between groups with differing number of awakenings was statistically significant (*F*_*(*2, 149)_ = 4.69, *p* = .011, *η²* = 0.06, 95% CI [0.00, 0.14]), with post-hoc tests revealing that PSQI scores were significantly higher, representing poorer sleep quality, in the group of carers who experienced > 2 awakenings per night than the carers who reported no overnight awakenings (*p* = .025, 95% CI [0.24, 4.62]). No statistically significant difference was detected between PSQI scores in carers who experienced 1–2 awakenings when compared to carers who reported no awakenings (*p* = .574, 95% CI [-1.29, 3.19]) or between PSQI scores in carers who experienced > 2 awakenings versus 1–2 awakenings (*p* = .057, 95% CI [-0.03, 2.99]). Sensitivity analyses revealed similar results (*F*_*(*2, 149)_ = 3.53, *p* = .032, *η²* =0.05) when using revised PSQI scores (omitting duration of sleep and sleep disturbance sub-scales). Post-hoc results were also similar for carers who experienced > 2 awakenings per night than the carers who reported no overnight awakenings (*p* = .042, 95% CI [0.05, 3.32]), carers who experienced 1–2 awakenings when compared to carers who reported no awakenings (*p* = .467, 95% CI [-0.84, 2.51]), and carers who experienced > 2 awakenings versus 1–2 awakenings (*p* = .181, 95% CI [-0.28, 1.98]). Overall, these findings indicate that H1.1 was partially supported, with poorer sleep quality observed primarily among carers experiencing more than two night-time awakenings.

After adjusting for age, gender, and the number of care-recipients, ANCOVA revealed a difference in PSQI scores (*F*_(2, 146)_ = 3.78, *p* = .025, η² = 0.05) as a function of the number of awakenings experienced by carers each night. Despite these findings, post-hoc comparisons with covariates showed no difference in sleep quality between carers who experienced no awakenings compared to 1–2 awakenings (*p* = .672, 95% CI [-1.45, 3.08]), no difference between those who experienced no awakenings compared to > 2 awakenings (*p* = .056, 95% CI [-0.042, 4.46]), and no difference between those who experienced 1–2 awakenings compared to > 2 awakenings (*p* = .082, 95% CI [-0.138, 2.93]). Despite the lack of differences, none of the covariates demonstrated statistical significance in the model. Sensitivity analyses revealed differences were no longer significant (*F*_(2, 146)_ = 2.60, *p* = .078, η² = 0.03) when using revised PSQI scores (omitting duration of sleep and sleep disturbance sub-scales). Therefore, H1.2 was not supported, as post-hoc comparisons did not demonstrate significant differences between awakening groups after adjustment for covariates.

After adjusting for assistive technology use and help-seeking behaviors, ANCOVA revealed a difference in PSQI scores (*F*_(2, 147)_ = 4.50, *p* = .013, η² = 0.06) as a function of the number of awakenings experienced by carers each night. Post-hoc comparisons with covariates showed a difference in sleep quality between carers who experienced no awakenings compared to > 2 awakenings (*p* = .026, 95% CI [0.24, 4.50]), yet no difference between carers who experienced no awakenings compared to 1–2 awakenings (*p* = .520, 95% CI [-1.16, 3.14]) or 1–2 awakenings compared to > 2 awakenings (*p* = .069, 95% CI [-0.08, 2.84]). Help-seeking behaviours, yet not assistive technology use, demonstrated statistical significance in the model. Sensitivity analyses revealed similar results (*F*_(2, 147)_ = 3.18, *p* = .044, η² = 0.04) when using revised PSQI scores (omitting duration of sleep and sleep disturbance sub-scales). Post-hoc results were also similar for carers who experienced 1–2 awakenings when compared to carers who reported no awakenings (*p* = .443, 95% CI [-0.80, 2.47]) and carers who experienced > 2 awakenings versus 1–2 awakenings (*p* = .233, 95% CI [-0.34, 1.87]), yet differences were no longer significance for carers who experienced > 2 awakenings per night versus carers who reported no overnight awakenings (*p* = .052, 95% CI [-0.01, 3.23]). Therefore, H1.3 was partially supported, with poorer sleep quality evident among carers experiencing more than two awakenings per night compared to those reporting no awakenings.

### H2 (assistive technology use and sleep quality)

Approximately half of all carers (49.2%) reported that they did not use any assistive technologies or devices. Of those that were used, pressure mattresses/bedding (12.2%), locks (11.5%), and temperature controls (11.5%) were the most commonly used. A summary of assistive technology use by carers is shown in Table 3.

**Table 3:** Use of assistive technologies or devices in Australian adult carers

Do you use any assistive technologies or devices?		Total (*n* = 262)	
		*n*	%
No		129	49.2
Yes	Pressure mattress/bedding	32	12.2
	Locks	30	11.5
	Temperature controls	30	11.5
	Other ^a^	29	11.1
	Personal alarm call system	27	10.3
	Blood glucose meter	26	9.9
	Video display/hands free phone	20	7.6
	Continuous glucose monitor	15	5.7
	Bell	14	5.3
	Care dog	12	4.6
	Insulin pump	12	4.6
	Location/movement monitoring system	10	3.8
	Epileptic seizure alarm	8	3.1
	Fall detector	7	2.7
	Enuresis monitors	1	0.4

Note: *n* Number of participants, % Percentage of participants. Participants were able to select all options that applied

^a^ Text responses for ‘Other” consisted of: mobile phone (n = 3, 1.2%), adjustable bed (n = 3, 1.2%), baby monitor (n = 2, 0.8%), walkie talkie (n = 2, 0.8%), sleep apnoea device (n = 2, 0.8%), pulse oximeter (n = 2, 0.8%) and haemodialysis alarm (n = 1, 0.4%). Further ‘Other’ responses did not identify the assistive technologies or devices which were used

This section examines whether assistive technology use is associated with carers’ sleep quality, both unadjusted and after accounting for demographic and care-related factors. Carers who reported using assistive technologies (*M* = 12.1, *SD* = 3.6) had slightly higher PSQI scores than carers who reported no assistive technology use (*M* = 11.9, *SD* = 3.9), but this relationship was not significant (t _(150)_ = -0.26, *p* = .797, Cohen’s d = − 0.04, 95% CI [-0.36, 0.28]). Therefore, H2.1 was not supported, as assistive technology use was not associated with better sleep quality.

After adjusting for age and gender, ANCOVA revealed no difference in PSQI scores (*F*_(1, 148)_ = 0.20, *p* = .653, *η²* = 0.001) based on greater use of assistive technology. Therefore, H2.2 was not supported. After adjusting for night-time awakenings and help-seeking behaviors, ANCOVA revealed no difference in PSQI scores (*F*_(1, 148)_ = 0.71, *p* = .400, η² = 0.005) based on greater use of assistive technology. Therefore, H2.3 was not supported.

### H3 (help-seeking behaviors and sleep quality)

**Fig. 2:**
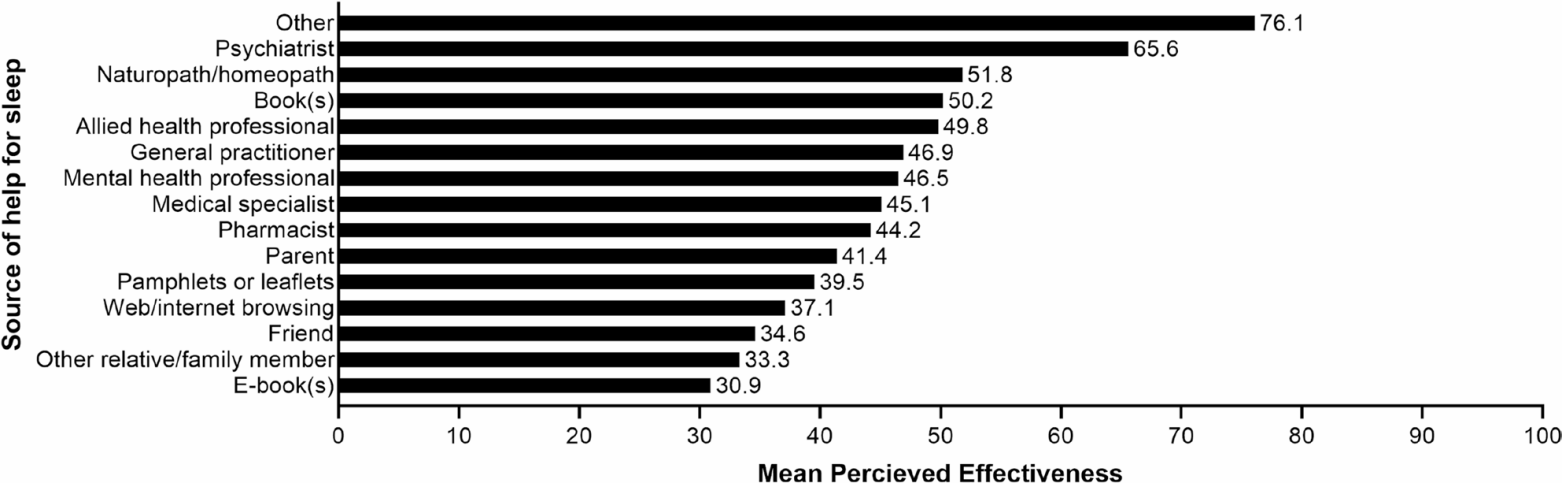
Mean perceived effectiveness of help-seeking behaviours for sleep problems in Australian carers. Values indicate mean perceived effectiveness of help-seeking behaviours as measured on a scale of 0 to 100, where 0 indicated not at all effective and 100 indicated extremely effective. Participants only rated the effectiveness of help-seeking behaviours which they had used within the past 12 months. Due to a survey error, data pertaining to the perceived effectiveness of help-seeking via an intimate partner was not collected

A summary of help-seeking behaviors is shown in Table 4 and perceived effectiveness of help-seeking is shown in Fig. 2. Approximately half of all carers (46.5%) reported that they had not sought help for their sleep problems in the past 12 months. For those that had, general practitioner (28.8%), mental health professional (21.5%), and web/internet browsing (14.2%), were the most commonly reported. The help-seeking behavior with the highest perceived effectiveness was the ‘Other’ option, with a mean effectiveness rating of 76.1 on a scale of 0 to 100; this included participating in a carer support group, sleep studies, dietician, pain medication, and sleep apps. Help-seeking from a general practitioner, which was the most common help-seeking behavior, was rated as moderately effective (46.9 on a scale of 0 to 100).

**Table 4:** Help-Seeking behaviours for sleep problems in Australian adult carers

In the past 12 months have you sought help for your sleep problems?		Total (*n* = 262)	
		*n*	%
No		121	46.5
Yes	General practitioner	75	28.8
	Mental health professional	56	21.5
	Web/internet browsing	37	14.2
	Intimate partner	28	10.8
	Pharmacist	23	8.8
	Friend	21	8.1
	Parent	19	7.3
	Allied health professional	14	5.4
	Naturopath/homeopath	13	5.0
	Book(s)	12	4.6
	Medical specialist ^a^	12	4.6
	Other relative/family member	11	4.2
	Psychiatrist	10	3.8
	E-book(s)	8	3.1
	Other ^b^	5	1.9
	Pamphlets or leaflets	4	1.5

Note: *n* Number of participants, % Percentage of participants. Participants were able to select all options that applied

^a^ Text responses for ‘Medical specialist’ consisted of sleep specialist (*n* = 3, 1.2%), ear nose and throat specialist (*n* = 2, 0.8%), sleep clinic (*n* = 2, 0.8%), sleep physician (*n* = 1, 0.4%), rheumatologist (*n* = 1, 0.4%), sleep endocrinologist (*n* = 1, 0.4%), and specialist in complex health problems (*n* = 1, 0.4%). One participant did not further specify the type of medical specialist they sought help from

^b^ Text responses for ‘Other’ consisted of carer support group (*n* = 1, 0.4%), sleep study (*n* = 1, 0.4%), dietician (*n* = 1, 0.4%), pain medication (*n* = 1, 0.4%), and sleep app (*n* = 1, 0.4%)

This section examines whether help-seeking behaviors are associated with carers’ sleep quality, before and after adjustment for relevant covariates. PSQI scores were higher in carers who reported help-seeking for their sleep problems (*M* = 12.9, *SD* = 3.7) when compared to carers who reported no help-seeking (*M* = 10.6, *SD* = 3.3). PSQI scores were significantly higher in carers who had sought help compared to those who had not (t (150) = 4.07, *p* < .001, Cohen’s d = 0.67, 95% CI [0.34, 1.01]). Post-hoc comparisons revealed that carers who had reported seeking help for their sleep problems had significantly poorer sleep quality than carers who had not sought help (*p* < .001, 95% CI [-3.54, -1.17]). Therefore, H3.1 was not supported, as greater help-seeking was associated with poorer, rather than better, sleep quality.

After adjusting for age and gender, ANCOVA revealed a difference in PSQI scores (*F*_(1, 148)_ = 15.36, *p* < .001, *η²* = 0.09) based on greater use of assistive technology. Therefore, H3.2 was not supported, with results indicating poorer sleep quality among carers who reported help-seeking.

After adjusting for night-time awakenings and help-seeking behaviors, ANCOVA revealed no difference in PSQI scores (*F*_(1, 148)_ = 16.05, *p* < .001, η² = 0.09) based on greater use of assistive technology. Post-hoc comparisons revealed that carers who had reported seeking help for their sleep problems had significantly poorer sleep quality than carers who had not sought help (*p* < .001, 95% CI [-3.48, -1.18]). Therefore, H3.3 was not supported, as carers who sought help continued to report poorer sleep quality.

## Discussion

The present study examined associations between night-time awakenings, assistive technology use, and help-seeking behaviors on self-reported sleep quality of adult carers who report inadequate sleep. Most participants reported poor subjective sleep quality, with those reporting > 2 awakenings per night having poorer sleep quality compared with carers who reported no awakenings. There was no statistical difference in sleep quality between carers who used assistive technology and those who did not. Carers who reported help-seeking for sleep problems reported poorer sleep quality compared to carers who had not sought help.

### Overnight awakenings

Relative to carers who did not report overnight awakenings, carers who reported > 2 awakenings per night had poorer sleep quality. While there was no statistical difference in the sleep quality of carers who reported 1–2 awakenings and that of other groups (0 awakenings, > 2 awakenings), raw data suggests a tendency for sleep quality to reduce as number of awakenings increases. This finding is consistent with a previous study of young carers aged 15–18 years, which found that carers who experienced > 2 awakenings had poorer sleep quality than those who reported < 2 awakenings [5].

Adult carers in the present study were four times more likely to report either 1–2 or > 2 awakenings compared to no awakenings. Despite this seemingly high frequency, literature suggests that the self-reported number of awakenings among carers are often underestimated. Previous research using objective sleep assessment (e.g., actigraphy) found carers woke, on average, 8 times overnight [30, 31]. However, it is important to consider potential differences in how awakenings are defined between subjective and objective sleep studies. Carers in the present study may have identified each time they woke to get out of bed, whereas objective sleep measures may identify awakenings that are not subjectively recalled. Actigraphy also predicts sleep and wake states from movement data and may, at times, overestimate awakenings [31]. Regardless of the tools used, the present and previous research shows that carers can experience frequent night-time awakenings [5, 11, 32], which can negatively affect overall sleep quality [5].

A notable limitation of the present study is the lack of data regarding the reasons underpinning carers’ awakenings. While the PSQI captured the number of awakenings, it did not determine whether these were due to caregiving responsibilities, personal sleep disturbances, or other environmental factors. Future research should aim to differentiate between caregiving-related awakenings and those unrelated to care demands, as the nature of the disruption may influence both sleep quality and the type of intervention needed. Identifying whether awakenings are necessary and care-driven or potentially preventable could inform more targeted support strategies for carers. Additionally, it is important to acknowledge that for many carers, night-time awakenings are an essential and non-negotiable aspect of providing care. In such cases, reducing the number of awakenings may not be realistic. Rather than aiming to eliminate these disruptions, future work should prioritise strategies that help carers maintain or improve overall sleep quality within the constraints of their caring role. This may include exploring interventions that promote restorative sleep during non-caregiving periods, improve sleep efficiency, or provide respite support to enable recovery sleep. A shift in focus from eliminating awakenings to supporting sleep despite them may offer more practical and meaningful outcomes for carers.

### Assistive technology use

The present study found no statistical difference in sleep quality between carers who used assistive technologies and those who did not. This finding was inconsistent with previous research which had suggested that assistive technology use overnight was associated with better self-reported sleep quality in carers [19]. It was hypothesised that, since assistive technology use had been associated with reduced carer burden and anxiety overnight, there would be positive effects on carer sleep quality [17, 33]. However, it has been acknowledged that sleep quality could be negatively impacted by assistive technology use overnight due to device alerts causing further sleep disruption [17, 19].

One potential reason for the lack of difference in sleep quality between those who used assistive technology and those who did not may be in that participants were asked to indicate any assistive technology use, rather than the use of devices *overnight*, as assistive technologies being used exclusively during the day would not necessarily impact sleep. Despite this, each of the assistive technology options could be used overnight, and all could potentially impact carers’ sleep, whether by directly reducing the number of times a carer would check on their care-recipient, or by assisting the care-recipient in a way that would reduce their need for care overnight [17]. Interestingly, carers who did not use assistive technology were almost three times more likely to experience no awakenings overnight, whereas carers who did use assistive technology were 1.4 times more likely to experience > 2 awakenings. While these findings could suggest that assistive technology use is associated with more awakenings, it is likely that both the use of assistive technology and increased number of awakenings may be due to higher care-recipient needs. A care-recipient with more complex needs may require both more assistive technology use, and more awakenings for care. It is important to consider that while assistive technology could lead to increased awakenings for carers, these awakenings may be appropriate and lead to improved health outcomes of their care-recipient [6]. Due to a lack of research exploring this relationship, further evidence is needed to determine how the use of assistive technologies overnight impacts the sleep of carers and whether any relationship is impacted by the nature of the care-recipient condition.

### Help-Seeking behaviors

It was anticipated that help-seeking for sleep problems would be associated with better sleep quality due to a reduction in carer burden [34]. Conversely, the present study found that help-seeking for sleep problems in carers was associated with poorer self-reported sleep quality. A possible explanation for this is that carers who have worse sleep outcomes are in-turn more likely to seek help for their sleep problems. This finding is similar to that of Attard et al. [35], who found that within the Australian general public, short sleep duration and sleep problems were predictors of help-seeking behaviors.

Concerningly, despite seeking help, carers are still presenting with poor quality sleep. Even more worrying was the low perceived effectiveness of all help-seeking options. Formal help-seeking from a general practitioner or mental health professional were the most common help-seeking options, with these sources rated below 50% effective in helping sleep problems. The highest-rated help-seeking option (apart from ‘other’) was seeing a psychiatrist, which was rated at 65.6% effectiveness. This finding is consistent with that of Chang et al. [36], who found that those experiencing sleep problems were the most willing to seek help from a psychiatrist compared to other help-seeking options. Despite many more participants indicating that they had sought help from a general practitioner, this option was rated as being less effective. This is an interesting finding considering that both psychiatrists and general practitioners can prescribe medications used for sleep problems, however, seeing a psychiatrist is perceived as being more effective. It is important to note that while there are medications available to assist with sleep problems, the use of sedative medications may not be a viable option for carers who may still have to wake during the night to provide care. It was also noted that almost half of all carers surveyed had not sought any help for their sleep problems. This is an alarming statistic considering that all participants had been identified as obtaining inadequate sleep. This finding, however, is consistent with previous research which suggests that carers may be hesitant to seek help for their own problems [25, 37, 38], as they do not want to be seen as not coping in their carer role [38]. It is important for the wellbeing of carers that they feel comfortable seeking help for themselves without feeling as if they are taking away from the medical attention that their care-recipient receives. Further research is needed to understand the relationship between help-seeking and the sleep quality of carers, with a focus on examining the barriers experienced by carers in seeking help for their own sleep problems.

### Strengths, limitations & future directions

The cross-sectional approach of this study carries methodological limitations which should be considered when interpreting the outcomes. First, this design precludes determination of causality [39], with future investigations benefiting from longitudinal designs to allow for more detailed examinations of causality between carer awakenings, assistive technology use, help-seeking behaviors, and sleep quality. Second, reliance on self-reported data could lead to inaccuracies. Although the PSQI is a validated tool, combining self-report measures with objective methods (e.g., actigraphy or polysomnography) could provide a more comprehensive and accurate assessment, reducing the risk of Type I error. The absence of objective measures and sleep diaries also limits insight into key behaviours such as daytime napping or sleep fragmentation, which may compensate for night-time awakenings but are not captured by the PSQI. Future research would benefit from integrating subjective and objective assessments and adopting prospective designs to better understand the dynamics between carer awakenings, assistive technology use, help-seeking behaviours, and overall sleep quality. Additionally, some overlap exists between PSQI items (e.g., sleep disturbances, duration) and the measure of night-time awakenings used in this study. This may have introduced shared variance, making it difficult to determine the unique impact of awakenings on overall sleep quality. Future research should consider using complementary tools to better isolate specific sleep parameters. Third, inclusion criteria limited participation to carers reporting inadequate sleep. This introduces a bias, and the inclusion of carers with adequate sleep in future research could aid in developing more effective sleep interventions or recommendations. Additionally, understanding how the severity of care-recipient needs affects the relationship between assistive technology use and carer sleep quality would be valuable. Longitudinal studies tracking changes in sleep quality before and after engagement with help-seeking strategies would also be of benefit. Given the low proportion of carers seeking help despite inadequate sleep, future research should explore barriers faced by carers in seeking help for their sleep. Finally, it must be acknowledged that convenience sampling in the broader study may have contributed to selection bias, with carers experiencing sleep problems more likely to engage in a project related to these challenges.

## Conclusion

Among carers reporting poor sleep, self-reported sleep quality was poorer in those providing night-time care more frequently. Carer who sought help for their own sleep problems also reported poorer sleep quality, when compared to those who have not sought help. Future research would benefit from objective measures of sleep quality, a prospective design, and broader eligibility criteria to further understand the relationship between carer awakenings, assistive technology use, help-seeking behaviors, and sleep quality.

NB: Violin plots showing the global PSQI score of carers across the number of self-reported awakenings. The width of each violin plot represents the approximate frequency of data located in each area. The boxplot within each violin plot shows how data is distributed. The horizontal line within each box plot represents the median value. The box represents the 25th to 75th percentiles and the vertical lines represent the 5th and 95th percentiles.

Values indicate mean perceived effectiveness of help-seeking behaviours as measured on a scale of 0 to 100, where 0 indicated not at all effective and 100 indicated extremely effective. Participants only rated the effectiveness of help-seeking behaviours which they had used within the past 12 months. Due to a survey error, data pertaining to the perceived effectiveness of help-seeking via an intimate partner was not collected.

## Supplementary Information

Supplementary Material 1

## Data Availability

The de-identified data that supports the findings of this study are available from the corresponding author upon reasonable request.
