# Supplementary material for: How do night-time awakenings, assistive technologies, and help-seeking behaviors impact the sleep of Australian carers? A cross-sectional study
Source: BMC Geriatr. 2026 Feb 9;26:342. doi: 10.1186/s12877-026-07024-6 (PMC12990515; doi:10.1186/s12877-026-07024-6)
Supplement: Supplementary file 1 — Supplementary Material 1 [file 12877_2026_7024_MOESM1_ESM.docx]

**Supplementary Material A**

STROBE Statement—checklist of items that should be included in reports of observational studies

|  | **Item No** | **Recommendation** | **Page(s)** |
| --- | --- | --- | --- |
| **Title and abstract** | 1 | (*a*) Indicate the study’s design with a commonly used term in the title or the abstract | 1 |
|  |  | (*b*) Provide in the abstract an informative and balanced summary of what was done and what was found | 2 |
| **Introduction** | | |  |
| Background/rationale | 2 | Explain the scientific background and rationale for the investigation being reported | 3-5 |
| Objectives | 3 | State specific objectives, including any prespecified hypotheses | 5 |
| **Methods** | | |  |
| Study design | 4 | Present key elements of study design early in the paper | 5 |
| Setting | 5 | Describe the setting, locations, and relevant dates, including periods of recruitment, exposure, follow-up, and data collection | 5-6 |
| Participants | 6 | (*a*) *Cohort study*—Give the eligibility criteria, and the sources and methods of selection of participants. Describe methods of follow-up  *Case-control study*—Give the eligibility criteria, and the sources and methods of case ascertainment and control selection. Give the rationale for the choice of cases and controls  *Cross-sectional study*—Give the eligibility criteria, and the sources and methods of selection of participants. | 5-6 |
|  |  | (*b*) *Cohort study*—For matched studies, give matching criteria and number of exposed and unexposed  *Case-control study*—For matched studies, give matching criteria and the number of controls per case | NA |
| Variables | 7 | Clearly define all outcomes, exposures, predictors, potential confounders, and effect modifiers. Give diagnostic criteria, if applicable | 6-8 |
| Data sources/ measurement | 8* | For each variable of interest, give sources of data and details of methods of assessment (measurement). Describe comparability of assessment methods if there is more than one group | 6-8 |
| Bias | 9 | Describe any efforts to address potential sources of bias | 6, 8-9 |
| Study size | 10 | Explain how the study size was arrived at | 5-6 |
| Quantitative variables | 11 | Explain how quantitative variables were handled in the analyses. If applicable, describe which groupings were chosen and why | 8-9 |
| Statistical methods | 12 | (*a*) Describe all statistical methods, including those used to control for confounding | 9 |
|  |  | (*b*) Describe any methods used to examine subgroups and interactions | 9 |
|  |  | (*c*) Explain how missing data were addressed | NA |
|  |  | (*d*) *Cohort study*—If applicable, explain how loss to follow-up was addressed  *Case-control study*—If applicable, explain how matching of cases and controls was addressed  *Cross-sectional study*—If applicable, describe analytical methods taking account of sampling strategy | NA |
|  |  | (*e*) Describe any sensitivity analyses | NA |

| **Results** | | |  |
| --- | --- | --- | --- |
| Participants | 13* | (a) Report numbers of individuals at each stage of study—eg numbers potentially eligible, examined for eligibility, confirmed eligible, included in the study, completing follow-up, and analysed | 9 |
|  |  | (b) Give reasons for non-participation at each stage | NA |
|  |  | (c) Consider use of a flow diagram | NA |
| Descriptive data | 14* | (a) Give characteristics of study participants (eg demographic, clinical, social) and information on exposures and potential confounders | 9-10 |
|  |  | (b) Indicate number of participants with missing data for each variable of interest | 9 |
|  |  | (c) *Cohort study*—Summarise follow-up time (eg, average and total amount) | NA |
| Outcome data | 15* | *Cohort study*—Report numbers of outcome events or summary measures over time | NA |
|  |  | *Case-control study—*Report numbers in each exposure category, or summary measures of exposure | NA |
|  |  | *Cross-sectional study—*Report numbers of outcome events or summary measures | 11-18 |
| Main results | 16 | (*a*) Give unadjusted estimates and, if applicable, confounder-adjusted estimates and their precision (eg, 95% confidence interval). Make clear which confounders were adjusted for and why they were included | 11-18 |
|  |  | (*b*) Report category boundaries when continuous variables were categorized | 8 |
|  |  | (*c*) If relevant, consider translating estimates of relative risk into absolute risk for a meaningful time period | NA |
| Other analyses | 17 | Report other analyses done—eg analyses of subgroups and interactions, and sensitivity analyses | NA |
| **Discussion** | | |  |
| Key results | 18 | Summarise key results with reference to study objectives | 19 |
| Limitations | 19 | Discuss limitations of the study, taking into account sources of potential bias or imprecision. Discuss both direction and magnitude of any potential bias | 23-24 |
| Interpretation | 20 | Give a cautious overall interpretation of results considering objectives, limitations, multiplicity of analyses, results from similar studies, and other relevant evidence | 19-23 |
| Generalisability | 21 | Discuss the generalisability (external validity) of the study results | 20-24 |
| **Other information** | | |  |
| Funding | 22 | Give the source of funding and the role of the funders for the present study and, if applicable, for the original study on which the present article is based | 5, 24 |

*Give information separately for cases and controls in case-control studies and, if applicable, for exposed and unexposed groups in cohort and cross-sectional studies.

**Note:** An Explanation and Elaboration article discusses each checklist item and gives methodological background and published examples of transparent reporting. The STROBE checklist is best used in conjunction with this article (freely available on the Web sites of PLoS Medicine at http://www.plosmedicine.org/, Annals of Internal Medicine at http://www.annals.org/, and Epidemiology at http://www.epidem.com/). Information on the STROBE Initiative is available at www.strobe-statement.org.

**Supplementary Material B**

**Understanding the sleep of Australian carers**

**Start of Block: Introduction**

Q1 **Understanding the sleep of Australian carers**   We are interested in understanding your current sleep habits and learning more about you and the person you care for. There are no right or wrong answers, please answer as honestly as you can so we can provide the best support and resources we can for you.   The aim of this survey is to find out about your sleep, and what strategies you might use to improve your sleep. We are trying to find the best ways to support carers. The information gathered from this survey will be used by Carers Australia and the Sleep Health Foundation to help improve the support carers receive.   Most people will be able to complete the survey within 15 minutes.

Q2 **Instructions:**   ·     Please try to answer all the questions. Work through the questions with someone else if needed (e.g. a family member or friend) ·     **You do not have to complete this questionnaire in one sitting.** Take your time and take breaks if you need to. You will be able to save your responses and come back to them later.  ·     If you are unsure about how to answer a question, please give the best answer you can, or indicate if not applicable. ·     If you feel you need support, please contact Lifeline on 13 11 14 or Beyond Blue on 1300 22 4636.   **To contact the research team please email a.reynolds@cqu.edu.au**

**End of Block: Introduction**

**Start of Block: Demographics_1**

Q3 What is your gender?

- Male (1)
- Female (2)
- Other (3)

Q4 How old are you?

▼ 14 or younger (4) ... 100 (92)

*Skip To: End of Survey If How old are you? = 14 or younger*

Q5
What best describes the area you live in?
 
 If you are not sure how to classify the area you live in, see this colour-coded map:

- Major city (1)
- Inner regional (2)
- Outer regional (3)
- Remote (4)
- Very remote (5)

| 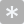 |
| --- |

Q6 What is your postcode?

________________________________________________________________

Q7 What was your employment status over the past 3 months? Were you...
*(select all that apply)*

- Working full-time (1)
- Working part-time (2)
- A full-time student (3)
- A part-time student (4)
- A homemaker (5)
- Unemployed (6)
- Retired (7)
- Unable to work due to disability (8)
- Volunteer (9)
- Other (please specify) (11) ________________________________________________

Q8 In the last 3 months, have you received the following government payments?
*(select all that apply)*

- Carer allowance / payment (1)
- Newstart / unemployment payment (4)
- Student allowance / payment (5)
- Age pension (6)
- Other government allowance / payment (please specify) (2) ________________________________________________
- ⊗No government payments received (3)

Q9 Please indicate the current members of your household (do not include yourself).

|  |  |
| --- | --- |
| ⊗Children aged 0 - 5 years (1) | ▼ 0 (1) ... 20 (23) |
| ⊗Children aged 6 - 10 years (2) | ▼ 0 (1) ... 20 (23) |
| ⊗Children aged 11 - 17 years (4) | ▼ 0 (1) ... 20 (23) |
| ⊗Adults aged 18 - 65 years (5) | ▼ 0 (1) ... 20 (23) |
| ⊗Adults aged 65+ years (6) | ▼ 0 (1) ... 20 (23) |

Q10 In general, would you say your health is:

- Excellent (1)
- Very good (2)
- Good (3)
- Fair (4)
- Poor (5)

**End of Block: Demographics_1**

**Start of Block: Carer Screening**

Q11 How many people do you currently provide assistance for (e.g. personal care, assistance with activities of daily living) because of their long-term illness, disability, or frailty? 


NOTE: This does not include healthy/typically developing children.

- 1 (1)
- 2 (2)
- 3 (3)
- 4 (4)
- More than 4 (5)
- I do not provide care (6)

*Skip To: End of Survey If How many people do you currently provide assistance for (e.g. personal care, assistance with acti... = I do not provide care*

**End of Block: Carer Screening**

**Start of Block: Sleep screening**

Q12 This section of the survey asks about how you manage your sleep

Q13 How SATISFIED/DISSATISFIED  are you with your CURRENT sleep pattern?

- Very satisfied (1)
- Satisfied (2)
- Moderately satisfied (3)
- Dissatisfied (4)
- Very dissatisfied (5)

Q14 How many hours of sleep do you regularly get each night?

- Less than 4 h (1)
- 4:00 - 4:59 h (2)
- 5:00 - 5:59 h (3)
- 6:00 - 6:59 h (4)
- 7:00 - 7:59 h (5)
- 8:00 - 8:59 h (6)
- 9 h or more (7)

Q15 Please list the top three strategies you use to manage your own sleep (i.e. things you to do get better sleep)


NOTE: If you do not use any strategies to manage your own sleep, please select 'I do not have any strategies to manage my sleep', below.

- Strategy 1 (1) ________________________________________________
- Strategy 2 (2) ________________________________________________
- Strategy 3 (3) ________________________________________________
- ⊗I do not have any strategies to manage my sleep (4)

**End of Block: Sleep screening**

**Start of Block: Sleep management strategies**

Q16 Which of the following do you currently use to manage your own sleep? 


(please select ALL that apply, including any you may have mentioned in the previous question)

- Napping (1)
- Going to bed early (2)
- Over-the-counter sleep medications (e.g. herbal supplements) (3)
- Prescription sleep medications (e.g. melatonin, benzodiazepines) (4)
- Sleep tracking technology (e.g. FitBit) (5)
- Using respite care services (i.e. government funded carers, paid nurses, etc.) (6)
- Having family and/or friends provide support (e.g. with bedtimes) (7)
- Having a consistent pre-bed routine (8)
- Getting a comfortable mattress and/or bedding (9)
- Altering eating or caffeine habits (10)
- Exercising (11)
- Sleep aids (e.g. eye mask, ear plugs) (12)
- Meditation/Relaxation/Yoga (13)
- Alcohol or other substances (14)
- Other (please specify) (15) ________________________________________________
- ⊗I do not use any strategies to manage my sleep (16)

*Display This Question:*

*If Which of the following do you currently use to manage your own sleep?  (please select ALL that ap... = Napping*

Q17 How effective do you find **napping** in managing your sleep?

|  | Not at all effective | Extremely effective |
| --- | --- | --- |

|  | 0 | 10 | 20 | 30 | 40 | 50 | 60 | 70 | 80 | 90 | 100 |
| --- | --- | --- | --- | --- | --- | --- | --- | --- | --- | --- | --- |

| () | 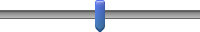 |
| --- | --- |

*Display This Question:*

*If Which of the following do you currently use to manage your own sleep?  (please select ALL that ap... = Going to bed early*

Q18 How effective do you find **going to bed early**in managing your sleep?

|  | Not at all effective | Extremely effective |
| --- | --- | --- |

|  | 0 | 10 | 20 | 30 | 40 | 50 | 60 | 70 | 80 | 90 | 100 |
| --- | --- | --- | --- | --- | --- | --- | --- | --- | --- | --- | --- |

| () | 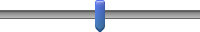 |
| --- | --- |

*Display This Question:*

*If Which of the following do you currently use to manage your own sleep?  (please select ALL that ap... = Over-the-counter sleep medications (e.g. herbal supplements)*

Q19 How effective do you find **over the counter medications**in managing your sleep?

|  | Not at all effective | Extremely effective |
| --- | --- | --- |

|  | 0 | 10 | 20 | 30 | 40 | 50 | 60 | 70 | 80 | 90 | 100 |
| --- | --- | --- | --- | --- | --- | --- | --- | --- | --- | --- | --- |

| () | 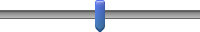 |
| --- | --- |

*Display This Question:*

*If Which of the following do you currently use to manage your own sleep?  (please select ALL that ap... = Prescription sleep medications (e.g. melatonin, benzodiazepines)*

Q20 How effective do you find**prescription medications**in managing your sleep?

|  | Not at all effective | Extremely effective |
| --- | --- | --- |

|  | 0 | 10 | 20 | 30 | 40 | 50 | 60 | 70 | 80 | 90 | 100 |
| --- | --- | --- | --- | --- | --- | --- | --- | --- | --- | --- | --- |

| () | 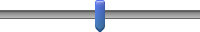 |
| --- | --- |

*Display This Question:*

*If Which of the following do you currently use to manage your own sleep?  (please select ALL that ap... = Sleep tracking technology (e.g. FitBit)*

Q21 How effective do you find**sleep tracking technology**in managing your sleep?

|  | Not at all effective | Extremely effective |
| --- | --- | --- |

|  | 0 | 10 | 20 | 30 | 40 | 50 | 60 | 70 | 80 | 90 | 100 |
| --- | --- | --- | --- | --- | --- | --- | --- | --- | --- | --- | --- |

| () | 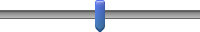 |
| --- | --- |

*Display This Question:*

*If Which of the following do you currently use to manage your own sleep?  (please select ALL that ap... = Using respite care services (i.e. government funded carers, paid nurses, etc.)*

Q22 How effective do you find**using respite services**in managing your sleep?

|  | Not at all effective | Extremely effective |
| --- | --- | --- |

|  | 0 | 10 | 20 | 30 | 40 | 50 | 60 | 70 | 80 | 90 | 100 |
| --- | --- | --- | --- | --- | --- | --- | --- | --- | --- | --- | --- |

| () | 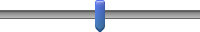 |
| --- | --- |

*Display This Question:*

*If Which of the following do you currently use to manage your own sleep?  (please select ALL that ap... = Having family and/or friends provide support (e.g. with bedtimes)*

Q23 How effective do you find**assistance from family/friends**in managing your sleep?

|  | Not at all effective | Extremely effective |
| --- | --- | --- |

|  | 0 | 10 | 20 | 30 | 40 | 50 | 60 | 70 | 80 | 90 | 100 |
| --- | --- | --- | --- | --- | --- | --- | --- | --- | --- | --- | --- |

| () | 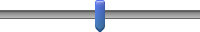 |
| --- | --- |

*Display This Question:*

*If Which of the following do you currently use to manage your own sleep?  (please select ALL that ap... = Having a consistent pre-bed routine*

Q24 How effective do you find**having a consistent pre-bed routine**in managing your sleep?

|  | Not at all effective | Extremely effective |
| --- | --- | --- |

|  | 0 | 10 | 20 | 30 | 40 | 50 | 60 | 70 | 80 | 90 | 100 |
| --- | --- | --- | --- | --- | --- | --- | --- | --- | --- | --- | --- |

| () | 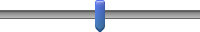 |
| --- | --- |

*Display This Question:*

*If Which of the following do you currently use to manage your own sleep?  (please select ALL that ap... = Getting a comfortable mattress and/or bedding*

Q25 How effective do you find**changing your bedding**in managing your sleep?

|  | Not at all effective | Extremely effective |
| --- | --- | --- |

|  | 0 | 10 | 20 | 30 | 40 | 50 | 60 | 70 | 80 | 90 | 100 |
| --- | --- | --- | --- | --- | --- | --- | --- | --- | --- | --- | --- |

| () | 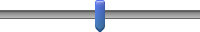 |
| --- | --- |

*Display This Question:*

*If Which of the following do you currently use to manage your own sleep?  (please select ALL that ap... = Altering eating or caffeine habits*

Q26 How effective do you find**altering your eating/caffeine habits**in managing your sleep?

|  | Not at all effective | Extremely effective |
| --- | --- | --- |

|  | 0 | 10 | 20 | 30 | 40 | 50 | 60 | 70 | 80 | 90 | 100 |
| --- | --- | --- | --- | --- | --- | --- | --- | --- | --- | --- | --- |

| () | 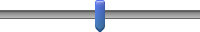 |
| --- | --- |

*Display This Question:*

*If Which of the following do you currently use to manage your own sleep?  (please select ALL that ap... = Exercising*

Q27 How effective do you find**exercising**in managing your sleep?

|  | Not at all effective | Extremely effective |
| --- | --- | --- |

|  | 0 | 10 | 20 | 30 | 40 | 50 | 60 | 70 | 80 | 90 | 100 |
| --- | --- | --- | --- | --- | --- | --- | --- | --- | --- | --- | --- |

| () | 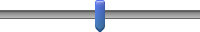 |
| --- | --- |

*Display This Question:*

*If Which of the following do you currently use to manage your own sleep?  (please select ALL that ap... = Sleep aids (e.g. eye mask, ear plugs)*

Q28 How effective do you find**sleep aids (e.g. eye mask, ear plugs)**in managing your sleep?

|  | Not at all effective | Extremely effective |
| --- | --- | --- |

|  | 0 | 10 | 20 | 30 | 40 | 50 | 60 | 70 | 80 | 90 | 100 |
| --- | --- | --- | --- | --- | --- | --- | --- | --- | --- | --- | --- |

| () | 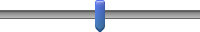 |
| --- | --- |

*Display This Question:*

*If Which of the following do you currently use to manage your own sleep?  (please select ALL that ap... = Meditation/Relaxation/Yoga*

Q29 How effective do you find**meditation, relaxation, or yoga**in managing your sleep?

|  | Not at all effective | Extremely effective |
| --- | --- | --- |

|  | 0 | 10 | 20 | 30 | 40 | 50 | 60 | 70 | 80 | 90 | 100 |
| --- | --- | --- | --- | --- | --- | --- | --- | --- | --- | --- | --- |

| () | 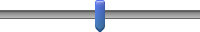 |
| --- | --- |

*Display This Question:*

*If Which of the following do you currently use to manage your own sleep?  (please select ALL that ap... = Alcohol or other substances*

Q30 How effective do you find**alcohol or other substances**in managing your sleep?

|  | Not at all effective | Extremely effective |
| --- | --- | --- |

|  | 0 | 10 | 20 | 30 | 40 | 50 | 60 | 70 | 80 | 90 | 100 |
| --- | --- | --- | --- | --- | --- | --- | --- | --- | --- | --- | --- |

| () | 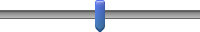 |
| --- | --- |

*Display This Question:*

*If Which of the following do you currently use to manage your own sleep?  (please select ALL that ap... = Other (please specify)*

Q31 How effective do you find**${Q16/ChoiceTextEntryValue/15}**in managing your sleep?

|  | Not at all effective | Extremely effective |
| --- | --- | --- |

|  | 0 | 10 | 20 | 30 | 40 | 50 | 60 | 70 | 80 | 90 | 100 |
| --- | --- | --- | --- | --- | --- | --- | --- | --- | --- | --- | --- |

| () | 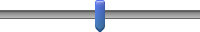 |
| --- | --- |

Q32 If you have anything else you would like to tell us about the strategies you use to manage your sleep, please do so here:

________________________________________________________________

________________________________________________________________

**End of Block: Sleep management strategies**

**Start of Block: Care recipient demographics**

Q33 **The next part of this survey is about the person you provide care for.**   *If you care for more than one person, for the purposes of this survey please think about* ***the person receiving care who is most disruptive to your sleep*** *(i.e. the care recipient who disturbs your sleep most, either physically due to disrupted sleep at night, or emotionally due to worry or concern).*

Q34 Which of the following best describes the health condition experienced by the care recipient? 

(please select all that apply)

- Acquired brain injury (1)
- Alzheimer's / dementia (2)
- Arthritis (3)
- Autism Spectrum Disorder (14)
- Back pain (4)
- Burns (15)
- Cancer (16)
- Cardiovascular disease (5)
- Chromosomal or syndromic conditions (e.g. Rett syndrome, Angelman syndrome) (6)
- Chronic respiratory conditions (7)
- Cystic fibrosis (8)
- Dermatological condition other than severe atopic dermatitis (17)
- Diabetes Mellitus Type 1 (18)
- Downs syndrome (9)
- Drug or alcohol problem (38)
- Epilepsy (10)
- Fragile X syndrome (11)
- Frail aged (39)
- Gastrointestinal conditions (19)
- Haematological conditions (12)
- HIV/AIDS (20)
- Immunodeficiency (21)
- Intellectual disability (22)
- Mental health conditions (e.g. schizophrenia, major depression) (23)
- Metabolic conditions treated by medically prescribed diet (e.g. urea cycle deficits) (25)
- Multiple sclerosis (26)
- Myalgic encephalitis / chronic fatigue syndrome (27)
- Neurodegenerative disorders (e.g. Ataxia Telangiectasia) (28)
- Neurometabolic degenerative conditions (e.g. Tay Sach's) (29)
- Neuromuscular conditions (e.g. Duchenne muscular dystrophy) (30)
- Organ failure (31)
- Osteogenesis Imperfecta (32)
- Phenylketonuria (PKU) (33)
- Physical or neurological disability impacting mobility (e.g. cerebral palsy, spina bifida) (34)
- Sensory impairments (e.g. blindness) (35)
- Severe atopic dermatitis (36)
- Ulcerative colitis (37)
- Other (please specify) (13) ________________________________________________

Q35 Do you believe that their condition is a lifelong condition or a condition from which they are expected to recover?

- Lifelong (1)
- I expect them to recover (2)

Q36 What age is the care recipient?

▼ < 1 year (4) ... 100+ (206)

Q37 What gender is the care recipient?

- Male (1)
- Female (2)
- Other (3)

**End of Block: Care recipient demographics**

**Start of Block: Impact of caring on your sleep**

Q38 **This section includes some more questions about how your caring role affects your sleep**

Q39 Please list the top three things about your caring role that impact your sleep?

- 1 (1) ________________________________________________
- 2 (2) ________________________________________________
- 3 (3) ________________________________________________

Q40 Which of the following aspects of your caring role or your personal circumstances results in poor sleep/sleep interruptions during the night?


(please select all that apply)

- Administering medication (1)
- Monitoring medical needs of your care recipient (e.g. checking medical status) (2)
- Monitoring medical equipment required by your care recipient (3)
- Worrying or rumination about the health or wellbeing of your care recipient (4)
- Being woken because your care recipient is distressed or engaging in challenging behavior (5)
- Putting your care recipient back to bed after a night time waking (6)
- Changing clothes/bedding due to care recipient incontinence/wetting the bed (7)
- Providing food/water for care recipient (8)
- Listening out for signs of waking/distress in care recipient (9)
- Your own health concerns (e.g. pain, medication needs) (10)
- Your own diagnosed sleep disorders (e.g. insomnia) (11)
- Other (please specify) (12) ________________________________________________

*Display This Question:*

*If Which of the following aspects of your caring role or your personal circumstances results in poor... = Administering medication*

Q41 To what extent does **administering medication** result in poor sleep/sleep interruptions for you?

|  | Not at all | A great deal |
| --- | --- | --- |

|  | 0 | 10 | 20 | 30 | 40 | 50 | 60 | 70 | 80 | 90 | 100 |
| --- | --- | --- | --- | --- | --- | --- | --- | --- | --- | --- | --- |

| () | 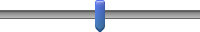 |
| --- | --- |

*Display This Question:*

*If Which of the following aspects of your caring role or your personal circumstances results in poor... = Monitoring medical needs of your care recipient (e.g. checking medical status)*

Q42 To what extent does **monitoring medical needs of your care recipient (e.g. checking medical status)** result in poor sleep/sleep interruptions for you?

|  | Not at all | A great deal |
| --- | --- | --- |

|  | 0 | 10 | 20 | 30 | 40 | 50 | 60 | 70 | 80 | 90 | 100 |
| --- | --- | --- | --- | --- | --- | --- | --- | --- | --- | --- | --- |

| () | 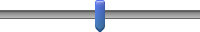 |
| --- | --- |

*Display This Question:*

*If Which of the following aspects of your caring role or your personal circumstances results in poor... = Monitoring medical equipment required by your care recipient*

Q43 To what extent does **monitoring medical equipment required by your care recipient** result in poor sleep/sleep interruptions for you?

|  | Not at all | A great deal |
| --- | --- | --- |

|  | 0 | 10 | 20 | 30 | 40 | 50 | 60 | 70 | 80 | 90 | 100 |
| --- | --- | --- | --- | --- | --- | --- | --- | --- | --- | --- | --- |

| () | 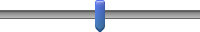 |
| --- | --- |

*Display This Question:*

*If Which of the following aspects of your caring role or your personal circumstances results in poor... = Worrying or rumination about the health or wellbeing of your care recipient*

Q44 To what extent does **worrying or rumination about the health or wellbeing of your care recipient** result in poor sleep/sleep interruptions for you?

|  | Not at all | A great deal |
| --- | --- | --- |

|  | 0 | 10 | 20 | 30 | 40 | 50 | 60 | 70 | 80 | 90 | 100 |
| --- | --- | --- | --- | --- | --- | --- | --- | --- | --- | --- | --- |

| () | 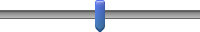 |
| --- | --- |

*Display This Question:*

*If Which of the following aspects of your caring role or your personal circumstances results in poor... = Being woken because your care recipient is distressed or engaging in challenging behavior*

Q45 To what extent does **being woken because your care recipient is distressed** result in poor sleep/sleep interruptions for you?

|  | Not at all | A great deal |
| --- | --- | --- |

|  | 0 | 10 | 20 | 30 | 40 | 50 | 60 | 70 | 80 | 90 | 100 |
| --- | --- | --- | --- | --- | --- | --- | --- | --- | --- | --- | --- |

| () | 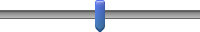 |
| --- | --- |

*Display This Question:*

*If Which of the following aspects of your caring role or your personal circumstances results in poor... = Putting your care recipient back to bed after a night time waking*

Q46 To what extent does **putting your care recipient back to bed after a night time waking** result in poor sleep/sleep interruptions for you?

|  | Not at all | A great deal |
| --- | --- | --- |

|  | 0 | 10 | 20 | 30 | 40 | 50 | 60 | 70 | 80 | 90 | 100 |
| --- | --- | --- | --- | --- | --- | --- | --- | --- | --- | --- | --- |

| () | 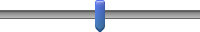 |
| --- | --- |

*Display This Question:*

*If Which of the following aspects of your caring role or your personal circumstances results in poor... = Changing clothes/bedding due to care recipient incontinence/wetting the bed*

Q47 To what extent does **changing clothes/bedding due to care recipient incontinence/wetting the bed**result in poor sleep/sleep interruptions for you?

|  | Not at all | A great deal |
| --- | --- | --- |

|  | 0 | 10 | 20 | 30 | 40 | 50 | 60 | 70 | 80 | 90 | 100 |
| --- | --- | --- | --- | --- | --- | --- | --- | --- | --- | --- | --- |

| () | 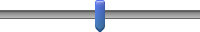 |
| --- | --- |

*Display This Question:*

*If Which of the following aspects of your caring role or your personal circumstances results in poor... = Providing food/water for care recipient*

Q48 To what extent does **providing food/water for care recipient**result in poor sleep/sleep interruptions for you?

|  | Not at all | A great deal |
| --- | --- | --- |

|  | 0 | 10 | 20 | 30 | 40 | 50 | 60 | 70 | 80 | 90 | 100 |
| --- | --- | --- | --- | --- | --- | --- | --- | --- | --- | --- | --- |

| () | 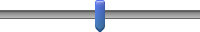 |
| --- | --- |

*Display This Question:*

*If Which of the following aspects of your caring role or your personal circumstances results in poor... = Listening out for signs of waking/distress in care recipient*

Q49 To what extent does **listening out for signs of waking/distress in care recipient**result in poor sleep/sleep interruptions for you?

|  | Not at all | A great deal |
| --- | --- | --- |

|  | 0 | 10 | 20 | 30 | 40 | 50 | 60 | 70 | 80 | 90 | 100 |
| --- | --- | --- | --- | --- | --- | --- | --- | --- | --- | --- | --- |

| () | 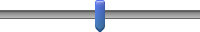 |
| --- | --- |

*Display This Question:*

*If Which of the following aspects of your caring role or your personal circumstances results in poor... = Your own health concerns (e.g. pain, medication needs)*

Q50 To what extent do **your own health concerns**result in poor sleep/sleep interruptions for you?

|  | Not at all | A great deal |
| --- | --- | --- |

|  | 0 | 10 | 20 | 30 | 40 | 50 | 60 | 70 | 80 | 90 | 100 |
| --- | --- | --- | --- | --- | --- | --- | --- | --- | --- | --- | --- |

| () | 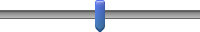 |
| --- | --- |

*Display This Question:*

*If Which of the following aspects of your caring role or your personal circumstances results in poor... = Your own diagnosed sleep disorders (e.g. insomnia)*

Q51 To what extent do **your own diagnosed sleep disorders**result in poor sleep/sleep interruptions for you?

|  | Not at all | A great deal |
| --- | --- | --- |

|  | 0 | 10 | 20 | 30 | 40 | 50 | 60 | 70 | 80 | 90 | 100 |
| --- | --- | --- | --- | --- | --- | --- | --- | --- | --- | --- | --- |

| () | 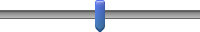 |
| --- | --- |

*Display This Question:*

*If Which of the following aspects of your caring role or your personal circumstances results in poor... = Other (please specify)*

Q52 To what extent do **${Q40/ChoiceTextEntryValue/12}**result in poor sleep/sleep interruptions for you?

|  | Not at all | A great deal |
| --- | --- | --- |

|  | 0 | 10 | 20 | 30 | 40 | 50 | 60 | 70 | 80 | 90 | 100 |
| --- | --- | --- | --- | --- | --- | --- | --- | --- | --- | --- | --- |

| () | 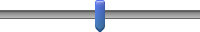 |
| --- | --- |

| 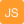 |
| --- |

Q53 On a typical night, how many times would you need to get out of bed to provide care?

▼ 0 (4) ... 30+ (62)

| 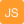 | 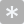 |
| --- | --- |

Q54 On a typical night, how many total minutes of care is required from the time you would typically go to sleep until the time you would typically wake up?


(i.e. any care requirements between 10:00pm - 7:00am if this is your typical sleep schedule)

________________________________________________________________

Q55 To what extent do you think your role as a carer interferes with your sleep **through caring for your care recipient**?

|  | Does not interfere at all | Extremely interfering |
| --- | --- | --- |

|  | 0 | 10 | 20 | 30 | 40 | 50 | 60 | 70 | 80 | 90 | 100 |
| --- | --- | --- | --- | --- | --- | --- | --- | --- | --- | --- | --- |

| () | 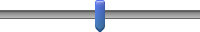 |
| --- | --- |

Q56 To what extent do you think your role as a carer interferes with your sleep through **worry or rumination** about your care recipient when you are trying to sleep?

|  | Does not interfere at all | Extremely interfering |
| --- | --- | --- |

|  | 0 | 10 | 20 | 30 | 40 | 50 | 60 | 70 | 80 | 90 | 100 |
| --- | --- | --- | --- | --- | --- | --- | --- | --- | --- | --- | --- |

| () | 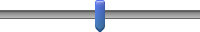 |
| --- | --- |

Q57 Do you use any of the following technologies or assistive devices?


(please select all that apply)

- Bell (1)
- Care dog (2)
- Insulin pump (3)
- Continuous glucose monitor (4)
- Blood glucose meter (5)
- Location/movement monitoring system (i.e. monitoring system that alerts caregiver when care recipient moves, leaves their bed, etc.) (6)
- Enuresis monitors (8)
- Locks (9)
- Temperature controls (12)
- Fall detector (14)
- Epileptic seizure alarm (15)
- Video display / hands free phone (16)
- Pressure mattress / bedding (17)
- Personal alarm call system (18)
- Other (please specify) (7) ________________________________________________
- ⊗I/we do not use any assistive technology (19)

*Display This Question:*

*If Do you use any of the following technologies or assistive devices? (please select all that apply) = Care dog*

Q58 Where does your care dog usually sleep?

- On the care recipient's bed (1)
- In the care recipient's room (not on their bed) (2)
- On your bed (3)
- In your room (not on your bed) (4)
- On the bed you and your care recipient share (5)
- In the room you and your care recipient share (not on the bed) (10)
- In another room (e.g. living room) (7)
- Outside (8)
- Other (please specify) (11) ________________________________________________

Q59 Is there any additional information you would like to provide about how your caring role impacts your sleep?

________________________________________________________________

________________________________________________________________

________________________________________________________________

________________________________________________________________

________________________________________________________________

**End of Block: Impact of caring on your sleep**

**Start of Block: Sleep behavior**

Q60 Do you share a bed or bedroom with your care recipient?

- Not during the past month (1)
- Less than once/week (2)
- Once or twice/week (3)
- Three or more times/week (4)
- Always (5)

Q61 Has your doctor or a clinician diagnosed you with a sleep disorder?

- Yes, obstructive sleep apnea (requiring treatment) (1)
- Yes, obstructive sleep apnea (NOT requiring treatment) (6)
- Yes, insomnia (2)
- Yes, restless legs/periodic limb movement disorder (3)
- Yes, other (please specify) (4) ________________________________________________
- ⊗No diagnosed sleep disorders (5)

*Display This Question:*

*If Has your doctor or a clinician diagnosed you with a sleep disorder? = Yes, obstructive sleep apnea (requiring treatment)*

Q62 What treatment do you receive for your obstructive sleep apnea?

- CPAP device (1)
- Diet (2)
- Exercise (3)
- Surgery (4)
- Other (please specify) (5) ________________________________________________
- ⊗No treatment (6)

*Display This Question:*

*If Has your doctor or a clinician diagnosed you with a sleep disorder? = Yes, insomnia*

Q63 What treatment do you receive for your insomnia?

- Prescription sleeping medication (e.g. benzodiazepines, melatonin) (1)
- Over the counter sleeping medication (e.g. herbal supplements) (2)
- Sleep education (i.e. education around your sleep habits or environment) (3)
- Bright light therapy (4)
- Exercise (5)
- Other (please specify) (6) ________________________________________________
- ⊗No treatment (7)

*Display This Question:*

*If Has your doctor or a clinician diagnosed you with a sleep disorder? = Yes, restless legs/periodic limb movement disorder*

Q64 What treatment do you receive for your restless legs/periodic limb movement disorder?

- Prescription sleeping medication (e.g. benzodiazepines, melatonin) (1)
- Over the counter sleeping medication (e.g. herbal supplements) (2)
- Exercise (3)
- Other (please specify) (4) ________________________________________________
- ⊗No treatment (5)

*Display This Question:*

*If Has your doctor or a clinician diagnosed you with a sleep disorder? = Yes, other (please specify)*

Q65 What treatment do you receive for your *other* sleep disorder?

- Prescription sleeping medication (e.g. benzodiazepines, melatonin) (1)
- Over the counter sleeping medication (e.g. herbal supplements) (2)
- Exercise (3)
- Sleep education (i.e. education around your sleep habits or environment) (4)
- Bright light therapy (5)
- Other (please specify) (6) ________________________________________________
- ⊗No treatment (7)

**End of Block: Sleep behavior**

**Start of Block: Sleep Hygiene Index**

Q66 I take daytime naps lasting two or more hours

- Always (1)
- Frequently (2)
- Sometimes (3)
- Rarely (4)
- Never (5)

Q67 I **go to bed** at different times from day to day

- Always (1)
- Frequently (2)
- Sometimes (3)
- Rarely (4)
- Never (5)

Q68 I **get out of bed** at different times from day to day

- Always (1)
- Frequently (2)
- Sometimes (3)
- Rarely (4)
- Never (5)

Q69 I exercise to the point of sweating within 1 hour of going to bed

- Always (1)
- Frequently (2)
- Sometimes (3)
- Rarely (4)
- Never (5)

Q70 I stay in bed longer than I should two or three times a week

- Always (1)
- Frequently (2)
- Sometimes (3)
- Rarely (4)
- Never (5)

Q71 I use alcohol, tobacco/e-cigarettes, or caffeine within 4 hours of going to bed or after going to bed

- Always (1)
- Frequently (2)
- Sometimes (3)
- Rarely (4)
- Never (5)

Q72 I do something that may wake me up before bedtime (e.g. play video games, use the internet, clean)

- Always (1)
- Frequently (2)
- Sometimes (3)
- Rarely (4)
- Never (5)

Q73 I go to bed feeling stressed, angry, upset or nervous

- Always (1)
- Frequently (2)
- Sometimes (3)
- Rarely (4)
- Never (5)

Q74 I use my bed for things other than sleeping or sex (e.g. watch television, read, eat, study)

- Always (1)
- Frequently (2)
- Sometimes (3)
- Rarely (4)
- Never (5)

Q75 I sleep on an uncomfortable bed (e.g. poor mattress or pillow, too much or not enough blankets)

- Always (1)
- Frequently (2)
- Sometimes (3)
- Rarely (4)
- Never (5)

Q76 I sleep in an uncomfortable bedroom (e.g. too bright, too stuffy, too hot, too cold, too noisy)

- Always (1)
- Frequently (2)
- Sometimes (3)
- Rarely (4)
- Never (5)

Q77 I do important work before bedtime (e.g. pay bills, schedule, or study)

- Always (1)
- Frequently (2)
- Sometimes (3)
- Rarely (4)
- Never (5)

Q78 I think, plan, or worry when I am in bed

- Always (1)
- Frequently (2)
- Sometimes (3)
- Rarely (4)
- Never (5)

**End of Block: Sleep Hygiene Index**

**Start of Block: PSQI**

Q79
**Only a few questions to go!**
  **The following questions relate to your usual sleep habits during the past month only.**
 
**Your answers should indicate the most accurate reply for the majority of days and nights in the past month.**

 **Please answer all questions.**

Q80 During the PAST MONTH, what time have you usually gone to bed at night?

|  | Hours | Minutes | AM/PM | |
| --- | --- | --- | --- | --- |
|  |  |  | AM (1) | PM (2) |
| Bed time (1) | ▼ 1 (1 ... 12 (12) | ▼ 00 (1 ... 45 (4) |  |  |

| 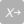 |
| --- |

Q81 During the PAST MONTH, how long has it usually taken you to fall asleep each night?

- 15 minutes or less (1)
- 16 - 30 minutes (2)
- 31 - 60 minutes (3)
- More than 60 minutes (4)

Q82
During the PAST MONTH, what time have you usually gotten up in the morning?

|  | Hours | Minutes | AM/PM | |
| --- | --- | --- | --- | --- |
|  |  |  | AM (1) | PM (2) |
| Get up time (1) | ▼ 1 (1 ... 12 (12) | ▼ 00 (1 ... 45 (4) |  |  |

Q83 During the **PAST MONTH**, how many hours of actual sleep did you usually get at night?
(This may be different than the number of hours you spent in bed.)

|  | Hours | Minutes |
| --- | --- | --- |
|  |  |  |
| Hours of sleep per night: (1) | ▼ 1 (1 ... 12 (12) | ▼ 00 (1 ... 45 (4) |

Q84 Is there any other reason(s) you have had trouble sleeping in the past month?

________________________________________________________________

| 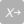 |
| --- |

Q85 During the past month, how often have you had trouble sleeping because of this?

|  | Not during the past month (1) | Less than once a week (2) | Once or twice a week (3) | Three or more times a week (4) | Not applicable (5) |
| --- | --- | --- | --- | --- | --- |
| Other reason (1) |  |  |  |  |  |

| 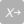 |
| --- |

Q86 During the past month, how would you rate your sleep quality overall?

- Very good (1)
- Fairly good (2)
- Fairly bad (3)
- Very bad (4)

| 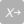 |
| --- |

Q87 During the past month...

|  | Not during the past month (1) | Less than once a week (2) | Once or twice a week (3) | Three or more times a week (4) |
| --- | --- | --- | --- | --- |
| How often have you taken medicine (prescribed or "over the counter") to help you sleep? (1) |  |  |  |  |
| How often have you had trouble staying awake while driving, eating meals, or engaging in social activity? (2) |  |  |  |  |

| 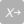 |
| --- |

Q88 During the past month, how much of a problem has it been for you to keep up enough enthusiasm to get things done?

- Not a problem at all (1)
- Only a very slight problem (2)
- Somewhat of a problem (3)
- A very big problem (4)

| 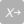 |
| --- |

Q89 During the past month, how often have you had trouble sleeping because you...

|  | Not during the past month (1) | Less than once a week (2) | Once or twice a week (3) | Three or more times a week (4) |
| --- | --- | --- | --- | --- |
| ...cannot get to sleep within 30 minutes (1) |  |  |  |  |
| ...wake up in the middle of the night or early morning (2) |  |  |  |  |
| ...have to get up to use the bathroom (3) |  |  |  |  |
| ...cannot breathe comfortably (4) |  |  |  |  |
| ...cough or snore loudly (5) |  |  |  |  |
| ...feel too cold (6) |  |  |  |  |
| ...feel too hot (7) |  |  |  |  |
| ...had bad dreams (8) |  |  |  |  |
| ...have pain (9) |  |  |  |  |

**End of Block: PSQI**

**Start of Block: Help seeking**

Q90 **The next few questions relate to any help you have received with your sleep.**

Q91 During the last 12 months, have you sought help with your sleep problems from any of the following?


(please select all that apply)

- ⊗I have not sought help for my sleep problems in the past 12 months (18)
- Intimate partner (e.g. girlfriend, boyfriend, spouse, de facto partner) (19)
- Friend (not related to you) (2)
- Parent (3)
- Other relative/family member (4)
- Mental health professional (e.g. psychologist, social worker, counsellor) (5)
- General practitioner (i.e. your doctor) (6)
- Allied health professional (e.g. physiotherapist, chiropractor) (7)
- Psychiatrist (8)
- Medical specialist (please specify) (9) ________________________________________________
- Pharmacist (10)
- Naturopath/homeopath (11)
- Web/internet browsing (12)
- E-book(s) (13)
- Book(s) (14)
- Pamphlets or leaflets from a local source (e.g. shopping centres, chemist) (15)
- Other (please specify) (16) ________________________________________________

*Display This Question:*

*If During the last 12 months, have you sought help with your sleep problems from any of the followin... =*

Q92 How effective did you find your intimate partner in helping your sleep problems?

|  | Not at all effective | Extremely effective |
| --- | --- | --- |

|  | 0 | 10 | 20 | 30 | 40 | 50 | 60 | 70 | 80 | 90 | 100 |
| --- | --- | --- | --- | --- | --- | --- | --- | --- | --- | --- | --- |

| () | 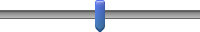 |
| --- | --- |

*Display This Question:*

*If During the last 12 months, have you sought help with your sleep problems from any of the followin... = Friend (not related to you)*

Q93 How effective did you find your friend in helping your sleep problems?

|  | Not at all effective | Extremely effective |
| --- | --- | --- |

|  | 0 | 10 | 20 | 30 | 40 | 50 | 60 | 70 | 80 | 90 | 100 |
| --- | --- | --- | --- | --- | --- | --- | --- | --- | --- | --- | --- |

| () | 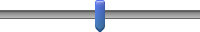 |
| --- | --- |

*Display This Question:*

*If During the last 12 months, have you sought help with your sleep problems from any of the followin... = Parent*

Q94 How effective did you find your parent in helping your sleep problems?

|  | Not at all effective | Extremely effective |
| --- | --- | --- |

|  | 0 | 10 | 20 | 30 | 40 | 50 | 60 | 70 | 80 | 90 | 100 |
| --- | --- | --- | --- | --- | --- | --- | --- | --- | --- | --- | --- |

| () | 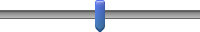 |
| --- | --- |

*Display This Question:*

*If During the last 12 months, have you sought help with your sleep problems from any of the followin... = Other relative/family member*

Q95 How effective did you find your other relative/family member in helping your sleep problems?

|  | Not at all effective | Extremely effective |
| --- | --- | --- |

|  | 0 | 10 | 20 | 30 | 40 | 50 | 60 | 70 | 80 | 90 | 100 |
| --- | --- | --- | --- | --- | --- | --- | --- | --- | --- | --- | --- |

| () | 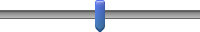 |
| --- | --- |

*Display This Question:*

*If During the last 12 months, have you sought help with your sleep problems from any of the followin... = Mental health professional (e.g. psychologist, social worker, counsellor)*

Q96 How effective did you find the mental health professional in helping your sleep problems?

|  | Not at all effective | Extremely effective |
| --- | --- | --- |

|  | 0 | 10 | 20 | 30 | 40 | 50 | 60 | 70 | 80 | 90 | 100 |
| --- | --- | --- | --- | --- | --- | --- | --- | --- | --- | --- | --- |

| () | 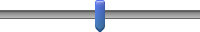 |
| --- | --- |

*Display This Question:*

*If During the last 12 months, have you sought help with your sleep problems from any of the followin... = General practitioner (i.e. your doctor)*

Q97 How effective did you find the general practitioner in helping your sleep problems?

|  | Not at all effective | Extremely effective |
| --- | --- | --- |

|  | 0 | 10 | 20 | 30 | 40 | 50 | 60 | 70 | 80 | 90 | 100 |
| --- | --- | --- | --- | --- | --- | --- | --- | --- | --- | --- | --- |

| () | 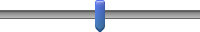 |
| --- | --- |

*Display This Question:*

*If During the last 12 months, have you sought help with your sleep problems from any of the followin... = Allied health professional (e.g. physiotherapist, chiropractor)*

Q98 How effective did you find the allied health professional in helping your sleep problems?

|  | Not at all effective | Extremely effective |
| --- | --- | --- |

|  | 0 | 10 | 20 | 30 | 40 | 50 | 60 | 70 | 80 | 90 | 100 |
| --- | --- | --- | --- | --- | --- | --- | --- | --- | --- | --- | --- |

| () | 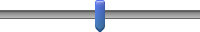 |
| --- | --- |

*Display This Question:*

*If During the last 12 months, have you sought help with your sleep problems from any of the followin... = Psychiatrist*

Q99 How effective did you find the psychiatrist in helping your sleep problems?

|  | Not at all effective | Extremely effective |
| --- | --- | --- |

|  | 0 | 10 | 20 | 30 | 40 | 50 | 60 | 70 | 80 | 90 | 100 |
| --- | --- | --- | --- | --- | --- | --- | --- | --- | --- | --- | --- |

| () | 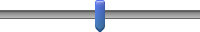 |
| --- | --- |

*Display This Question:*

*If During the last 12 months, have you sought help with your sleep problems from any of the followin... = Medical specialist (please specify)*

Q100 How effective did you find the medical specialist in helping your sleep problems?

|  | Not at all effective | Extremely effective |
| --- | --- | --- |

|  | 0 | 10 | 20 | 30 | 40 | 50 | 60 | 70 | 80 | 90 | 100 |
| --- | --- | --- | --- | --- | --- | --- | --- | --- | --- | --- | --- |

| () | 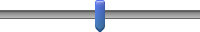 |
| --- | --- |

*Display This Question:*

*If During the last 12 months, have you sought help with your sleep problems from any of the followin... = Pharmacist*

Q101 How effective did you find the pharmacist in helping your sleep problems?

|  | Not at all effective | Extremely effective |
| --- | --- | --- |

|  | 0 | 10 | 20 | 30 | 40 | 50 | 60 | 70 | 80 | 90 | 100 |
| --- | --- | --- | --- | --- | --- | --- | --- | --- | --- | --- | --- |

| () | 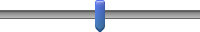 |
| --- | --- |

*Display This Question:*

*If During the last 12 months, have you sought help with your sleep problems from any of the followin... = Naturopath/homeopath*

Q102 How effective did you find the naturopath/homeopath in helping your sleep problems?

|  | Not at all effective | Extremely effective |
| --- | --- | --- |

|  | 0 | 10 | 20 | 30 | 40 | 50 | 60 | 70 | 80 | 90 | 100 |
| --- | --- | --- | --- | --- | --- | --- | --- | --- | --- | --- | --- |

| () | 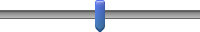 |
| --- | --- |

*Display This Question:*

*If During the last 12 months, have you sought help with your sleep problems from any of the followin... = Web/internet browsing*

Q103 How effective did you find internet browsing in helping your sleep problems?

|  | Not at all effective | Extremely effective |
| --- | --- | --- |

|  | 0 | 10 | 20 | 30 | 40 | 50 | 60 | 70 | 80 | 90 | 100 |
| --- | --- | --- | --- | --- | --- | --- | --- | --- | --- | --- | --- |

| () | 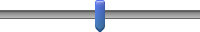 |
| --- | --- |

*Display This Question:*

*If During the last 12 months, have you sought help with your sleep problems from any of the followin... = E-book(s)*

Q104 How effective did you find reading e-book(s) in helping your sleep problems?

|  | Not at all effective | Extremely effective |
| --- | --- | --- |

|  | 0 | 10 | 20 | 30 | 40 | 50 | 60 | 70 | 80 | 90 | 100 |
| --- | --- | --- | --- | --- | --- | --- | --- | --- | --- | --- | --- |

| () | 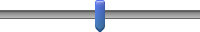 |
| --- | --- |

*Display This Question:*

*If During the last 12 months, have you sought help with your sleep problems from any of the followin... = Book(s)*

Q105 How effective did you find reading book(s) in helping your sleep problems?

|  | Not at all effective | Extremely effective |
| --- | --- | --- |

|  | 0 | 10 | 20 | 30 | 40 | 50 | 60 | 70 | 80 | 90 | 100 |
| --- | --- | --- | --- | --- | --- | --- | --- | --- | --- | --- | --- |

| () | 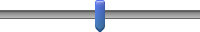 |
| --- | --- |

*Display This Question:*

*If During the last 12 months, have you sought help with your sleep problems from any of the followin... = Pamphlets or leaflets from a local source (e.g. shopping centres, chemist)*

Q106 How effective did you find reading pamphlets/leaflets in helping your sleep problems?

|  | Not at all effective | Extremely effective |
| --- | --- | --- |

|  | 0 | 10 | 20 | 30 | 40 | 50 | 60 | 70 | 80 | 90 | 100 |
| --- | --- | --- | --- | --- | --- | --- | --- | --- | --- | --- | --- |

| () | 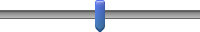 |
| --- | --- |

*Display This Question:*

*If During the last 12 months, have you sought help with your sleep problems from any of the followin... = Other (please specify)*

Q107 How effective did you find **${Q91/ChoiceTextEntryValue/16}** in helping your sleep problems?

|  | Not at all effective | Extremely effective |
| --- | --- | --- |

|  | 0 | 100 |
| --- | --- | --- |

| () | 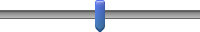 |
| --- | --- |

Q108 Do you have any additional comments you would like to share regarding the help you have sought or received with your sleep?

________________________________________________________________

________________________________________________________________

**End of Block: Help seeking**

**Start of Block: Demographics**

Q109 **The final few questions ask about your personal circumstances. This will help us to identify what kind of support carers may find helpful in future.**

Q110 What kind of respite services do you currently receive?

- ⊗No respite services are currently used (12)
- Short-term in-patient respite care (i.e. your care recipient will stay at a facility for a week or two at a time) (13)
- In home care support (i.e. a nurse or carer visits the care recipient’s home) for less than 5 h / week (2)
- In home care support (i.e. a nurse or carer visits the care recipient’s home) for 6 - 15 h / week (3)
- In home care support (i.e. a nurse or carer visits the care recipient’s home) for 16 - 30 h / week (4)
- In home care support (i.e. a nurse or carer visits the care recipient’s home) for more than 30 h / week (5)
- Care assistance out of home (e.g. day clubs) (6)
- Overnight respite in a care facility (7)
- Cottage respite (14)
- House cleaning services (8)
- Cooking support (e.g. meals on wheels, a personal chef) (9)
- Other (please specify) (15) ________________________________________________
- ⊗My care recipient currently lives in a residential care facility (e.g. hospital, nursing home, etc.) (10)

*Display This Question:*

*If What kind of respite services do you currently receive? != My care recipient currently lives in a residential care facility (e.g. hospital, nursing home, etc.)*

Q111 What living arrangement do you have with the person(s) you provide care for?

- We live in the same household all of the time (1)
- We live in the same household some of the time (2)
- My care recipient lives with another friend or relative (i.e. not in a care facility/hospital) (3)
- Other (please specify) (4) ________________________________________________

*Display This Question:*

*If What kind of respite services do you currently receive? != My care recipient currently lives in a residential care facility (e.g. hospital, nursing home, etc.)*

Q112 Are you considering residential care facilities for the person you provide care for?

- Not at all (1)
- Possibly in the next 5 years (2)
- Possibly in the next 12 months (3)
- Definitely, awaiting placement (4)

Q113 Below are statements that people have made about their standard of living. 


Please indicate how true these statements are for you using the scales below.

|  | Not true for me at all | Definitely true for me |
| --- | --- | --- |

|  | 0 | 10 | 20 | 30 | 40 | 50 | 60 | 70 | 80 | 90 | 100 |
| --- | --- | --- | --- | --- | --- | --- | --- | --- | --- | --- | --- |

| I can afford to go to a medical specialist if I need to () | 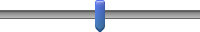 |
| --- | --- |
| I am able to visit people whenever I wish () | 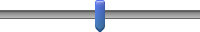 |
| I am able to give others as much as I want () | 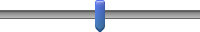 |
| I am able to do all the things I love () | 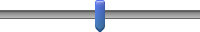 |
| I expect a future without money problems () | 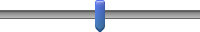 |
| My choices are limited by money () | 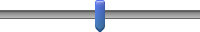 |

Q114 If you have anything else you would like to tell us about your role as a carer, please do so here:

________________________________________________________________

________________________________________________________________

________________________________________________________________

________________________________________________________________

________________________________________________________________

**End of Block: Demographics**
